# Incidence of diabetes mellitus following hospitalisation with influenza: a population-based cohort study in England

**DOI:** 10.1136/bmjopen-2025-115391

**Published:** 2026-05-04

**Authors:** Sophie Middleton, Tricia M McKeever, Frances S Grudzinska, Yue Huang, Charlotte E Bolton

**Affiliations:** 1 NIHR Nottingham Biomedical Research Centre, Nottingham, UK; 2 Centre for Respiratory Research, Translational Medical Sciences, University of Nottingham School of Medicine, Nottingham, England, UK; 3 Centre for Academic Primary Care, Lifespan and population Health, University of Nottingham School of Medicine, Nottingham, England, UK

**Keywords:** Respiratory infections, General diabetes, Hospitalization

## Abstract

**Abstract:**

**Objectives:**

To establish the incidence of developing diabetes mellitus (DM) post hospitalisation with influenza.

**Design:**

Retrospective cohort study.

**Setting:**

Electronic healthcare records from Clinical Practice Research Datalink (CPRD) linked to Hospital Episode Statistics in England.

**Participants:**

13 710 adults with a first episode of hospitalised influenza as the primary cause for admission between 1 July 2004 and 1 March 2021 based on ICD-10 codes without pre-existing DM were included. A randomly selected group (a) from CPRD records matched for age, sex and General Practitioner (GP) practice and (b) an unmatched group of hospitalised sepsis patients were used as comparator groups.

**Outcome measures:**

Patients were followed from 1 day after discharge till either DM diagnosis, death or end of GP record. HRs for incidence of DM were calculated using adjusted Cox regression models.

**Results:**

Incidence of DM was 12.5 per 1000 person years. Adjusted HRs (aHR) for developing DM after hospitalised influenza compared with matched controls was 1.54 (95% CI 1.39 to 1.70, p<0.001) and to hospitalised sepsis comparators 1.14 (95% CI 1.03 to 1.26, p=0.013). The greatest risk for developing DM in hospitalised influenza patients was within 90 days of discharge (aHR 2.71 (95% CI 1.94 to 3.77, p<0.001)) compared with matched controls. Risk factors for DM after influenza hospitalisation included being male, pre-existing DM risk, obesity and critical care admission during acute illness.

**Conclusion:**

Patients’ post hospitalisation with influenza had a greater incidence of DM when compared with both matched controls and patients following hospitalisation with sepsis.

**STRENGTHS AND LIMITATIONS OF THIS STUDY**

- Large sample size from linking two large, validated databases in England.
- Matched control group and a comparator group of hospitalised sepsis patients enabling control for confounding factors.
- Retrospective study using databases which rely on accuracy and timeliness of coding in primary care and secondary care.
- This study was unable to access information on the medical management of patients in hospital, such as corticosteroid use, which may influence development of diabetes after hospital admission.

## Introduction

 Influenza is a common acute respiratory illness (ARI), causing over 40 000 influenza-related hospital admissions in the UK every year,^1^ with an average healthcare cost of £30–£144 million per influenza season.^2^ Along with pneumonia, influenza is the most common reason for respiratory-related hospital admissions^3^ and is a core contributor to annual NHS winter pressures.^4^ The majority of patients survive acute illness and hospitalisation,^5^ but persistent symptoms, impaired exercise capacity, and raised inflammatory markers^6^ are reported more than 4 weeks following acute infection.^7^ However, little is known about the longer term health complications that may develop after hospitalisation with influenza.

The relationship between infection and diabetes is complex. It is known that people with hyperglycaemia and diabetes have increased risk of severe influenza infection and mortality.^8^ Conversely, ARI has been associated with new-onset diabetes, most notably in recent years after COVID-19 infection.^9^ Proposed underlying biological mechanisms include increased insulin resistance^10^ caused by stress hormones and cytokines due to persistent host inflammatory response; and direct infection and destruction of pancreatic beta cells causing a decrease in insulin production.^8 11^

Historically, during periods with increased influenza-like illness, there were increased admissions with diabetic ketoacidosis.^12^ Influenza animal models have demonstrated dysregulated glucose metabolism via impaired insulin signalling in liver tissues^13^ and influenza virus invasion and damage to the pancreas causing hyperglycaemia, but it is unclear how this translates clinically.^14 15^ Previous studies have postulated a link between pandemic influenza or a history of a preceding influenza-like illness and new onset type 1 diabetes in children^16^ and adults.^17 18^ Recent American studies have since demonstrated an increased risk of type 2 diabetes after influenza hospitalisation.^19 20^ It is unknown what the incidence of diabetes is after hospitalisation with influenza in England. This study aimed to estimate the incidence and severity of diabetes mellitus (DM) following hospitalisation with influenza in England, to compare this risk with non-hospitalised controls and with patients hospitalised with sepsis and to identify associated risk factors.

## Methods

### Source population

This cohort study was conducted using hospitalisation data from Hospital Episode Statistics (HES) linked to Clinical Practice Research Datalink (CPRD) Aurum. CPRD contains longitudinal data from anonymised electronic healthcare records in general practice.^21^ HES admitted patient care contains details of all admissions to NHS hospitals in England.^22^

### Study population and follow-up

Adults aged ≥18 years with a first episode of hospitalised influenza as the primary cause for admission between 1 July 2004 and March 2021 as recorded in CPRD linked to HES based on ICD-10 codes (J09-J11.8) (online supplemental table 1) on hospital discharge were included. Patients were excluded if they (1) had pre-existing DM or DM diagnosed during hospital admission, (2) died during hospital admission, (3) had hospital acquired influenza defined as hospitalisation in the prior 14 days from index date, (4) had a discharge date <1 year before GP practice registration date and (5) did not meet ‘acceptable’ data criteria in CPRD. CPRD labels healthcare records acceptable if they are deemed to be ‘research-quality’.

Patients were followed up from 1 day after discharge until the first of (1) the date of the patient’s death, (2) the date of the last collection of the practice, (3) the date of patient transferred out of the practice, (4) the date of the first Systematized Nomenclature of Medicine- Clinical Terms (SNOMED-CT) coded outcome of interest (5) or the end of the dataset.

### Control and comparator groups

Up to five randomly selected people with no prior diagnosis of DM and without hospitalisation with influenza in CPRD were matched with each patient admitted with pneumonia. They were matched for age (±1 year) at index date, sex and General Practitioner (GP) practice.

To consider the effect of being hospitalised with an inflammatory condition, a second unmatched hospitalised comparator group included adults ≥18 years old with a first episode of hospitalised sepsis as the primary cause for admission between 1 July 2004 till March 2021 as recorded in CPRD linked to HES based on ICD-10 codes (online supplemental table 1). Patients in this group had the same exclusion criteria as our study population but were also excluded if they had sepsis secondary to influenza or influenza concurrently diagnosed in the admission as per ICD10 codes. Patients in this group who had subsequent influenza admissions were also excluded.

### Outcomes

The primary outcome of interest was diagnosis of any DM. Secondary outcome was the diagnosis of prediabetic state (see below). Code lists for CPRD were compiled using the CPRD code browser application and manual search of SNOMED CT codes within CPRD. These were verified by another clinician. Code lists used are available on request.

Prediabetes was defined according to National Institute for Health and Care Excellence (NICE) Public Health Guideline PH38 (2012),^23^ incorporating impaired fasting glucose, impaired glucose tolerance and HbA1c 6%–6.4% as well as SNOMED codes for prediabetes.^23^ During analysis of prediabetes incidence, patients with pre-existing prediabetes prior to hospital admission were excluded. In patients who had a diagnosis of both DM and prediabetes during follow-up, prediabetes cases were only included if occurred prior to DM diagnosis.

### Covariate definitions

Medical comorbidities (prediabetes, chronic respiratory disease, hyperlipidaemia, hypertension) were defined using SNOMED CT code lists. For full definitions of covariates, please see online supplemental material.

A patient was classed as being ‘at risk of diabetes’ if they had a diagnosis of one of the above prediabetes diagnoses or fasting glucose or HbA1c measurements (closest to their admission date), indicating prediabetes, previous gestational diabetes or a code stating they were ‘at risk of diabetes’ prior to admission.

Prescription data were collected from CPRD, and pre-existing code lists used for oral corticosteroid (OCS), oral diabetic medication, insulin and influenza vaccination and updated for recent drug formulations. Prescriptions for OCS from 1 year prior to admission date to end of follow-up date were collected. Influenza vaccination was recorded if received in 1 year prior to admission date.

Testing data for hyperglycaemia and diabetes was collected from CPRD using a code list containing codes for random glucose tests, fasting glucose, glucose tolerance testing or HbA1c. The number of tests performed was noted for the year after hospital discharge or until diabetes diagnosis. A histogram was performed on the number of tests performed and people were categorised as a low tester if 0–1 tests were performed in the first year after discharge and a high tester if more than 1.

### Statistical analysis

All data management and statistical analysis were performed using R Statistical Software (V.4.5.1; R Core Team 2025). Descriptive statistics for the patient population were calculated. The baseline characteristics between people hospitalised for influenza and controls were compared by unpaired t test for parametric continuous data and χ^2^ for categorical data.

Time to diagnosis was calculated from the first day after hospital discharge till first diagnosis of diabetes or prediabetes in the GP records and absolute incidence rates (per 1000 person years) of development of diabetes calculated. When calculating incidence rates of prediabetes, patients with an active diagnosis of prediabetes or the most recent prediabetes measurement in prediabetic range prior to admission were excluded.

HRs for outcome measures were calculated using Cox regression models and aHR for potential confounders. Age at index date and gender were a priori confounders. Other potential confounders included body mass index (BMI), ethnicity, Indices of Multiple Deprivation (IMD) level, smoking status, pre-existing hypertension, pre-existing hyperlipidaemia, previous risk of diabetes, pre-existing chronic lung disease, critical care admission, oral steroid prescription 1 year prior to admission and during follow-up and influenza vaccination status in year prior to hospital admission. Each of the potential confounding variables was added into the model, one at a time, and were included in the model if they altered the HR by 5% or more. Missing data were present in BMI, ethnicity, smoking status and IMD level and were assumed as missing at random and imputed using multiple imputation model with five imputations (mice: Multivariate Imputation by Chained Equations in R, V.2011).

## Results

### Baseline characteristics

**Figure 1:**
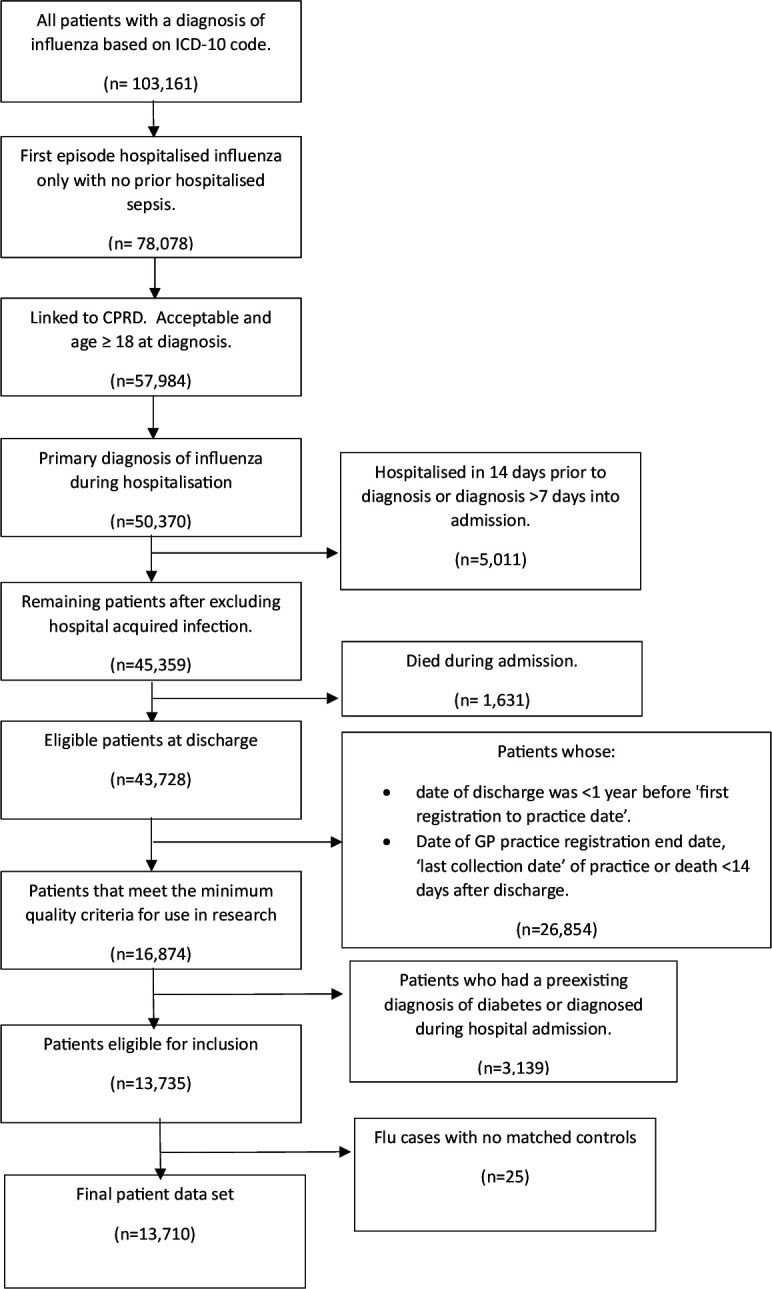
Flowchart of influenza study population selection. CPRD, Clinical Practice Research Datalink. GP, General Practitioner; ICD-10, International Classification of Diseases, 10th Revision.

After inclusion and exclusion criteria were applied (figure 1), the influenza study cohort comprised 13 710 patients with 42.5% male, mean (SD) age 57.6 (21.2)-year old and mean BMI 27.3 (6.5) kg/m^2^ (table 1). There were 61 384 and 38 561 patients included in the non-hospitalised matched controls and hospitalised sepsis comparator cohort, respectively. Non-hospitalised controls had a lower average BMI and were less likely to have any chronic pre-existing illness than hospitalised influenza patients. Patients in the hospitalised sepsis comparator cohort were older, more likely to be male, less likely to have pre-existing lung disease and had a longer length of hospitalisation than the influenza patients.

**Table 1:** Patient demographics at time of patient admission or matched cases time of admission in NH control group

	Influenza cohort	Non-hospitalised controls	P value*	Hospitalised sepsis comparators	P value†
N	13 710	61 384		38 561	
Age (mean, SD) years	57.6 (21.2)	55.8 (21.1)		67.5 (18.5)	<0.001
Male (**%**)	42.5	41.7	0.087	48.6	<0.001
Ethnicity, n (**%**)			<0.001		<0.001
Black	470 (3.4)	1944 (3.2)		781 (2.0)	
Mixed	152 (1.1)	733 (1.2)		238 (0.6)	
Other	430 (3.1)	1731 (2.8)		550 (1.4)	
South Asian	853 (6.2)	2636 (4.3)		923 (2.4)	
White	9852 (71.9)	43 801 (71.4)		24 390 (63.3)	
Missing	1953 (14.2)	10 539 (17.2)		11 679 (30.3)	
IMD (quintile) n(**%**)			<0.001		<0.001
Most deprived	3477 (25.4)	13 209 (21.5)		7549 (19.6)	
	2945 (21.5)	11 747 (19.1)		7460 (19.3)	
	2569 (18.7)	11 439 (18.6)		7537 (19.5)	
	2466 (18.0)	11 412 (18.6)		7954 (20.6)	
Least deprived	2241 (16.3)	11 192 (18.2)		8017 (20.8)	
Missing	12 (0.1)	2385 (3.9)		44 (0.1)	
BMI (kg/m^2^) (mean, SD)	27.3 (6.5)	26.8 (5.6)	<0.001	27.3 (6.4)	0.646
Missing n(**%**)	1369 (10.0)	8643 (14.1)		7946 (20.6)	
BMI class n(**%**)			<0.001		<0.001
<18.5 kg/m^2^	555 (4.0)	1431 (2.3)		1160 (3.0)	
18.5–24.9 kg/m^2^	4488 (32.7)	20 599 (33.6)		10 870 (28.2)	
25.0–29.9 kg/m^2^	3882 (28.3)	18 301 (29.8)		10 449 (27.1)	
>30 kg/m^2^	3416 (24.9)	12 410 (20.2)		8136 (21.1)	
Missing	1369 (10.0)	8643 (14.1)		7746 (20.6)	
Smoking status n(**%**)			<0.001		<0.001
Never	3723 (27.2)	19 537 (31.8)		9071 (23.5)	
Former	7330 (53.5)	30 220 (49.2)		20 362 (52.8)	
Current	2524 (18.4)	9808 (16.0)		4309 (11.2)	
Missing	133 (1.0)	1819 (3.0)		4819 (12.5)	
Chronic respiratory disease total n (**%**)	4462 (32.5)	9398 (15.3)	<0.001	5984 (15.5)	<0.001
‘At risk diabetes’ n(**%**)	1883 (13.7)	6189 (10.1)	<0.001	5286 (13.7)	0.939
Hyperlipidaemia n(**%**)	1439 (10.5)	5975 (9.7)	0.010	4678 (12.1)	<0.001
Hypertension n(**%**)	4109 (30.0)	15 214 (24.8)	<0.001	14 235 (36.9)	<0.001
Length of hospital stay days (median (IQR)	3 (1-7)	N/A		6 (3-13)	<0.001‡
Critical care admission (N**, %**)	933 (6.8)	N/A		2256 (5.9)	<0.001
≥1 OCS prescription (n,%)§	4793 (35.0)	7522 (12.3)	<0.001	9806 (25.4)	<0.001
Influenza vaccine status¶	2545 (18.6)	10 057 (16.4)	<0.001	8817 (22.9)	<0.001

* Comparison of non-hospitalised controls and influenza cohort.

† Comparison of hospitalised sepsis comparators and influenza cohort.

‡ Mann-Whitney U test.

§ Any prescription for OCS from 1 year prior to admission date to end of follow-up date included.

¶ Within a year prior to admission.

BMI, body mass index; IMD, Indices of Multiple Deprivation; NH, non-hospitalised; OCS, oral corticosteroid.

### Risk of diabetes mellitus

The crude incidence of developing DM was 12.5 per 1000 person years following influenza hospitalisation, 6.5 per 1000 person years in non-hospitalised controls and 11.7 per 1000 person years following sepsis hospitalisation. Unadjusted HR for developing DM compared with non-hospitalised controls was 1.91 (95% CI 1.74 to 2.10, p<0.001), (table 2). After adjustment for confounders the risk of developing any DM compared with NH controls remained significantly higher in the influenza population (1.54 (95% CI 1.39 to 1.70, p<0.001)). Only 12 cases of T1DM were identified post hospitalisation with influenza, therefore subgroup analysis of DM type was not performed.

**Table 2:** HRs for development of diabetes mellitus (DM) and prediabetes at follow-up after hospitalisation with influenza compared with matched non-hospitalised (NH) controls

	Cohort	Number of new diagnoses	Unadjusted HR (95% CI)	Adjusted HR (95% CI)	P value* (adjusted)
Diabetes Mellitus	Influenza cases	610	1.91 (1.74 to 2.10)	1.54 (1.39 to 1.70)†	<0.001
	NH controls	1591	1.0	1.0	
Prediabetes	Influenza cases N=12 044	721	1.46 (1.34 to 1.58)	1.28 (1.17 to 1.39)‡	<0.001
	NH controls N=55 915	2524	1.0	1.0	

* P value from likelihood ratio test.

† Adjusted for age at case index date, gender, BMI, preexisting hypertension, previous chronic lung disease, previous risk of diabetes, steroid prescription.

‡ Adjusted for age at case index date, gender, preexisting hypertension, previous chronic lung disease, steroid prescription.

BMI, body mass index.

In comparison to the hospitalised sepsis comparators, hospitalised influenza patients had an increased risk of developing DM (table 3). The adjusted HR for developing DM was 1.14 (95% CI 1.03 to 1.26, p=0.013) in the influenza group.

**Table 3:** HRs for development of diabetes mellitus (DM) and prediabetes at follow-up after hospitalisation with influenza compared with hospitalised sepsis patients

	Cohort	Number of new diagnoses	Unadjusted HR (95% CI)	Adjusted HR (95% CI)	P value* (adjusted)
Diabetes Mellitus	Influenza cases	610	1.08 (0.98 to 1.19)	1.14 (1.03 to 1.26)†	0.013
	Sepsis comparators	1258	1.0	1.0	
Prediabetes	Influenza cases N=12 044	721	1.13 (1.03 to 1.23)	1.29 (1.17 to 1.42)‡	<0.001
	Sepsis comparators N=33 735	1392	1.0	1.0	

* P value from likelihood ratio test.

† Adjusted for index date, gender, preexisting hypertension, previous chronic lung disease, steroid prescription.

‡ Adjusted for index date, gender, ethnicity, preexisting hypertension, previous chronic lung disease.

### Risk of prediabetes

After excluding patients with pre-existing prediabetes prior to admission 12 044 influenza patients, 55 915 matched controls and 33 735 sepsis patients remained with crude incidence of prediabetes development of 16.57 per 1000 person years, 11.32 per 1000 person years and 14.72 per 1000 person years, respectively (tables 2 and 3). After adjustment for confounders, there was a significant increased risk of developing prediabetes in the hospitalised influenza cohort as compared with the non-hospitalised, matched controls (aHR 1.28 (95% CI 1.17 to 1.39), p<0.001)) (table 2) and compared with hospitalised sepsis patients (aHR 1.29 (95% CI 1.17 to 1.42, p<0.001)) (table 3).

### Time to diagnosis of DM

The greatest risk for developing DM in hospitalised influenza patients was within 90 days of discharge (aHR 2.71 (95% CI 1.94 to 3.77, p<0.001)) compared with non-hospitalised, matched controls after adjustment for age, gender, BMI, oral steroid prescription, pre-existing hypertension, risk of diabetes and chronic lung disease. This difference within 90 days of discharge was not apparent between influenza and sepsis patients (aHR 1.14 (95% CI 0.85 to 1.51, p=0.383)) after adjustment for age and gender.

### Risk factors for DM development after influenza admission

Inherent characteristics such as male sex (aHR 1.22 (95% CI 1.07 to 1.34, p=0.002)) and age between 51 and 60-year old, compared with patients ≤40 years (aHR 2.86 (95% CI 2.12 to 3.86, p<0.001)) were risk factors for development of DM after influenza hospitalisation (online supplemental table 3). Modifiable risk factors such as increased BMI, hypertension and receiving a prescription for OCSs in the year before hospital admission or during the follow-up period, were associated with an increased risk of developing diabetes mellitus. Those admitted to critical care during their influenza admission were more likely to develop DM aHR 1.61 (95% CI 1.26 to 2.06, p<0.001).

In a subanalysis of influenza patients with a pre-existing diagnosis of prediabetes (N=1666), 215 developed DM during follow-up, 35% of all DM diagnoses. They were more likely to develop DM after influenza hospitalisation than those without pre-existing prediabetes (AHR 3.84, (95% CI 3.20 to 4.61, p<0.001)).

### Testing rate for hyperglycaemia and DM

More post hospitalisation influenza patients received at least one test for hyperglycaemia or DM in the first year after discharge compared with non-hospitalised, matched controls and sepsis comparators, 4728 (34.5%), 17 871 (29.1%) and 10 559 (27.4%) people respectively (p<0.001).

Irrespective of whether patients were tested 0–1 times in the first year after discharge (low testers) or tested more than once (high tester), influenza patients were more likely to be diagnosed with DM than matched NH controls (online supplemental table 4). In comparison with the hospitalised sepsis comparators, there was no difference in the rate of developing DM compared with influenza patients in ‘low-testers’ or ‘high testers’ (online supplemental table 5).

### Severity of DM

In the hospitalised influenza group, out of the 610 people who were diagnosed with diabetes during follow-up, 418 (68.5%) received either oral diabetic medication or insulin during the total follow-up period. This was a similar proportion as those hospitalised sepsis patients (846 (67.2%)) with new DM, and NH matched controls (995 (62.5%)) with new DM.

## Discussion

This is the first study in England to investigate the risk of DM after hospitalisation with influenza. The incidence of DM was markedly higher in those discharged from hospital after influenza than the estimated background incidence of T2DM in adults in England of 4.17 (95% CI 4.15 to 4.19) per 1000 person years.^24^

In contrast to our findings, a study of US veterans hospitalised for at least 5 days with influenza reported a T2DM incidence of 8.48 cases per 100 person-years.^19^ Importantly, however, this included people who developed diabetes both during hospitalisation and following discharge. Another US study showed that 11.2% of hospitalised influenza patients developed new-onset T2DM by 3-month follow-up. Both these populations included people who would have been at higher risk of diabetes generally.^25^ In context, post-COVID-19 hospitalisation DM incidence rates from the National Diabetes Audit (NDA) in the UK were 16.4 per 1000 population,^25^ rates more comparable to the findings in this study. A global meta-analysis of 9 studies after COVID-19 hospitalisation showed an incidence of DM of 15.53 cases per 1000 person-years of follow-up, although heterogeneity was high (I^2^=100%) and incidence rate ranged from 3.15 to 42.31 per 1000 person years.^26^ While increased rates of DM are consistently observed following hospitalisation for ARI, there is a variation in incidence across studies that likely reflects differences in population risk profiles and study design.

Those who developed DM after influenza hospitalisation were predominantly individuals with pre-existing risk factors for the condition.^27 28^ This is consistent with previous studies that demonstrated those with increasing age, increased BMI, and in-hospital hyperglycaemia were at increased risk of DM after influenza hospitalisation.^20^ Patients who were already at a higher risk of developing type 2 diabetes were also at increased risk of developing DM after hospitalisation with COVID-19^29^ and those of south Asian ethnicity.^9 25^ Higher risk of developing diabetes after COVID-19 infection was also noted in black ethnic groups and those living in more deprived socioeconomic areas,^9 25^ which was not seen in our study. The identification of high-risk individuals presents an opportunity for targeted influenza prevention and proactive monitoring for hyperglycaemia during and after hospitalisation.

Acute illness or injury can trigger hyperglycaemia, insulin resistance and glucose intolerance, often referred to as stress hyperglycaemia.^30^,^32^ However, the risk of developing DM after influenza appears to extend beyond this transient response. Similar patterns have been observed in hospitalised pneumonia patients, where individuals admitted with moderate to severe hyperglycaemia and no prior DM history were 2.99 (95% CI 2.07 to 4.31) to 11.43 (95% CI 7.50 to 17.42) times more likely to develop diabetes over 5 years compared with those with normoglycaemia.^33^ In a small influenza study, 46.2% of patients who developed DM in hospital continued to have DM at 3-month follow-up, a higher proportion than in a comparable COVID-19 cohort (46.2% vs 37%, p=0.0035).^20^ In our study, the majority of patients diagnosed with DM after influenza hospitalisation subsequently needed medical management for their diabetes. This implies a longer acting mechanism than the initial stress hyperglycaemia that leaves patients at risk of developing DM for months after their initial hospitalisation/illness that requires treatment.

A key strength of this study is the large sample size from linking 2 large, validated databases in England. There was a large, matched control group and a comparator group of hospitalised sepsis patients, so we were able to control for confounding factors such as being hospitalised and significant comorbidities. The sepsis comparator group was chosen as a potential similar patient population, hospitalised with an inflammatory condition although not every variable aligned, and notably, the length of stay was higher in this comparator group. The limitations in this study included reliance on accuracy and timeliness of coding in primary care and secondary care. Thus, the date the diagnosis was coded in the medical records may have been different to the date it was diagnosed. Although significant efforts are made by CPRD to ensure data quality and matching of linked data, we cannot fully exclude coding errors or missing data. Influenza patients who were not diagnosed with diabetes during follow-up were more likely to have missing data in their CPRD record than those that were. However, in further sensitivity analysis of ‘low testers’ or lower healthcare users, influenza patients were still more likely to be diagnosed with DM than NH controls. Due to the nature of HES data, we were unable to see medical management of patients in hospital, such as corticosteroid use, which may influence development of diabetes after hospital admission.

## Conclusion

Patients hospitalised with influenza had a greater incidence of DM when compared with both matched non-hospitalised controls and patients following hospitalisation with sepsis. This risk of DM was greatest within the first 90 days of admission, and the majority of patients required ongoing medical management. The longer term effects of influenza infection strengthen the case for better influenza prevention and surveillance to identify those with new onset DM.

## Supplementary material

10.1136/bmjopen-2025-115391 online supplemental file 1

## Data Availability

Data may be obtained from a third party and are not publicly available.
