# Supplementary material for: Incidence of diabetes mellitus following hospitalisation with influenza: a population-based cohort study in England
Source: BMJ Open. 2026 May 4;16(5):e115391. doi: 10.1136/bmjopen-2025-115391 (PMC13141003; doi:10.1136/bmjopen-2025-115391)
Supplement: online supplemental file 1 [file bmjopen-16-5-s001.docx]

**Incidence of Diabetes Mellitus Following Hospitalisation with Influenza: A population-based cohort study in England.**

**Supplementary material**

Sophie Middleton, Tricia M McKeever, Frances S Grudzinska, Yue Huang, Charlotte E Bolton

[Supplementary Table 1- ICD 10 codes for influenza 2](#_Toc213250050)

[Supplementary Table 2: ICD-10 codes for sepsis 2](#_Toc213250051)

Supplementary Table 3: Risk of new onset diabetes mellitus after hospitalisation with influenza stratified by risk factors for developing diabetes at time of admission. 4

Supplementary Table 4: Odds of developing any Diabetes Mellitus in the 1st year following discharge in influenza patients and non-hospitalised(NH) controls grouped by testing rate.................................................................................................................6 Supplementary Table 5: Odds of developing any Diabetes Mellitus in the 1st year following discharge in influenza patients and sepsis comparators grouped by testing rate…………………………………………………………………………………………………………………7

Supplementary Table 1- ICD 10 codes for influenza

| ICD 10 code | Definition |
| --- | --- |
| J09 | Influenza due to certain identified influenza virus |
| J10 | Influenza due to other identified influenza virus |
| J11 | Influenza, virus not identified |

Supplementary Table 2: ICD-10 codes for sepsis

| ICD 10 code | Definition |
| --- | --- |
| A02.1 | Salmonella sepsis |
| A20.7 | Septicaemic plague |
| A22.7 | Anthrax sepsis |
| A26.7 | Erysipelothrix sepsis |
| A32.7 | Listerial sepsis |
| A40 | Streptococcal sepsis |
| - A40.0 | Sepsis due to streptococcus, group A |
| - A40.1 | Sepsis due to streptococcus, group B |
| - A40.2 | Sepsis due to streptococcus, group D |
| - A40.3 | Sepsis due to Streptococcus pneumoniae |
| - A40.8 | Other streptococcal sepsis |
| - A40.9 | Streptococcal sepsis, unspecified |
| A41 | Other sepsis |
| - A41.0 | Sepsis due to Staphylococcus aureus |
| - A41.1 | Sepsis due to other specified staphylococcus |
| - A41.2 | Sepsis due to unspecified staphylococcus |
| - A41.3 | Sepsis due to Haemophilus influenzae |
| - A41.4 | Sepsis due to anaerobes |
| - A41.5 | Sepsis due to other Gram-negative organisms |
| - A41.8 | Other specified sepsis |
| - A41.9 | Sepsis, unspecified |
| A42.7 | Actinomycotic sepsis |
| B37.7 | Candidal sepsis |
| R57.2 | Septic shock |

**Supplementary material: Covariate definitions**

Ethnicity was defined as per CPRD data with ‘South Asian’ grouping including patients of ‘Indian’, ‘Pakistani’ and ‘Bangladeshi’ classifications in the dataset. Codelists were used for body mass index (BMI), smoking status and medical comorbidities (prediabetes, chronic respiratory disease, hyperlipidaemia, hypertension).

BMI was either calculated from recorded weight and height measurements or directly extracted from CPRD using observations prior to admission date. The closest recorded BMI to hospital discharge when aged over 18 years was used and then classified into BMI categories for analysis. Outlying values of BMI less than 10 or >100 kg/m^2^ were excluded.

Smoking status was taken as the most recent code prior to hospital admission. Those that had ever been coded as smoking but were now classed as non-smokers were reclassed as former smokers. Patients were classified as having a medical comorbidity if it was coded as a problem prior to admission.

Chronic respiratory disease was defined as having a current diagnosis of either asthma, chronic obstructive pulmonary disease, bronchiectasis or interstitial lung disease at time of admission.

Supplementary Table 3: Risk of new onset diabetes mellitus after hospitalisation with influenza stratified by risk factors for developing diabetes at time of admission.

|  |  | Influenza patients (n=13,710) | | |  |
| --- | --- | --- | --- | --- | --- |
| Predictive Variables | **New diabetes mellitus** | **Rate per 1000 person years** | **Unadjusted Hazard Ratio (95% CI)** | **Adjusted Hazard Ratio* (95% CI)** | **p-value**** |
| Age (years) | | | | | |
| 18-40 | 66 | 4.28 | 1.0 | 1.0 |  |
| 41-50 | 97 | 13.2 | 3.10 (2.27-4.24) | **2.31 (1.69-3.17)** | **<0.001** |
| 51-60 | 155 | 20.74 | 4.76 (3.57-6.35) | **2.86 (2.12-3.86)** | **<0.001** |
| 61-70 | 129 | 19.06 | 4.30 (3.19-5.80) | **2.39 (1.74-3.29)** | **<0.001** |
| 71-80 | 101 | 16.38 | 3.62 (2.64-4.94) | **2.00 (1.42-2.81)** | **<0.001** |
| >80 | 62 | 10.71 | 2.28 (1.61-3.24) | 1.35 (0.92-1.98) | 0.128 |
| Gender | | | | | |
| Male | 293 | 14.16 | 1.0 | 1.0 |  |
| Female | 317 | 11.21 | 0.79 (0.67-0.93) | **0.78 (0.66-0.93)** | **0.002** |
| Ethnicity | | | | | |
| White | 496 | 12.31 | 1.0 |  |  |
| Black | 24 | 11.23 | 0.95 (0.63-1.44) |  |  |
| Mixed | 10 | 14.79 | 1.16 (0.61-2.21) |  |  |
| Other | 26 | 13.42 | 1.08 (0.72-1.62) |  |  |
| South Asian | 54 | 13.74 | 1.17 (0.87-1.57) |  |  |
| IMD (Quintile) | | | | | |
| Most deprived | 178 | 14.24 | 1.39 (1.07-1.81) |  |  |
|  | 157 | 14.78 | 1.44 (1.10-1.88) |  |  |
|  | 95 | 10.37 | 1.01 (0.75-1.35) |  |  |
|  | 98 | 11.29 | 1.09 (0.82-1.47) |  |  |
| Least deprived | 82 | 10.24 | 1.0 |  |  |
| BMI class | | | | | |
| <18.5 kg/m^2^ | 6 | 3.09 | 0.63 (0.25-1.58) | 0.64 (0.26-1.62) | 0.34 |
| 18.5-24.9 kg/m^2^ | 93 | 5.16 | 1.0 | 1.0 |  |
| 25.0-29.9 kg/m^2^ | 178 | 11.51 | 2.09 (1.55-2.80) | **1.74 (1.29-2.33** | **<0.001** |
| >30 kg/m^2^ | 333 | 24.58 | 4.45 (3.41-5.82) | **3.13 (2.38-4.11)** | **<0.001** |
| Smoking status | | | | | |
| Never | 121 | 8.75 | 1.0 |  |  |
| Former | 371 | 14.46 | 1.64 (1.34-2.02) |  |  |
| Current | 118 | 12.45 | 1.43 (1.11-1.85) |  |  |
| Chronic Respiratory disease | | | | | |
| No | 379 | 11.13 | 1.0 |  |  |
| Yes | 231 | 15.48 | 1.36 (1.16-1.60) |  |  |
| At risk Diabetes | | | | | |
| No | 380 | 8.65 | 1.0 | 1.0 |  |
| Yes | 230 | 45.38 | 5.12 (4.33-6.05) | **3.77 (3.15-4.51)** | **<0.001** |
| Hyperlipidaemia | | | | | |
| No | 515 | 11.55 | 1.0 |  |  |
| Yes | 95 | 21.7 | 1.82 (1.46-2.27) |  |  |
| Hypertension | | | | | |
| No | 361 | 9.76 | 1.0 | 1.0 |  |
| Yes | 249 | 20.78 | 2.05 (1.74-2.42) | **1.21 (1.01-1.46)** | **0.037** |
| Critical care admission | | | | | |
| No | 536 | 11.82 | 1.0 | 1.0 |  |
| Yes | 74 | 20.33 | 1.75 (1.37-2.23) | **1.61 (1.26-2.06)** | **<0.001** |
| OCS prescription+ | | | | | |
| 0 | 293 | 9.45 | 1.0 | 1.0 |  |
| ≥1 | 317 | 17.63 | 1.89 (1.61-2.21) | **1.53 (1.30-1.80)** | **<0.001** |
| Influenza vaccine status^++^ | | | | | |
| No | 510 | 12.09 | 1.0 |  |  |
| Yes | 100 | 14.75 | 1.14 (0.92-1.42) |  |  |

IMD=Index of Multiple Deprivation, BMI=Body Mass Index, OCS=oral corticosteroid. Medical comorbidities present at index date.

* Adjusted for Age at index date, gender, BMI, preexisting hypertension, previous risk of diabetes, critical care admission, and steroid prescription.

** P Value from likelihood ratio test

+calculated from a year prior to admission to end of follow-up.

++ Within a year prior to admission

*Supplementary Table 4: Risk of new onset Diabetes Mellitus in the 1st year following discharge in influenza patients and non-hospitalised (NH) controls grouped by testing rate.*

|  | N (% of population) | N with diabetes diagnosed in 1^st^ year (%) | Unadjusted Hazard Ratio (95% CI) | Adjusted Hazard Ratio (95% CI) | P value |
| --- | --- | --- | --- | --- | --- |
| Low testers | | | | | |
| Influenza | 11,453 (83.5) | 73 (0.6) | 2.40 (1.82-3.18) | 1.99 (1.48- 2.65) ^a^ | <0.001 |
| NH controls | 53,808 (87.7) | 148 (0.3) | 1.0 | 1.0 |  |
| High testers | | | | | |
| Influenza | 2,257(16.4) | 120 (5.3) | 1.65 (1.33-2.06) | 1.56 (1.25-1.94) ^b^ | <0.001 |
| NH Controls | 7,576 (12.3) | 249 (3.3) | 1.0 | 1.0 |  |

*Low tester= 0-1 tests for diabetes/year, High tester= >1 test for diabetes per year*

1. *Adjusted for Age at index date, gender, BMI, IMD level, preexisting hypertension, preexisting chronic lung disease, previous risk of diabetes, and steroid prescription.*
2. *Adjusted for Age at index date, gender, BMI.*

*Supplementary Table 5: Risk of new onset Diabetes Mellitus in the 1st year following discharge in influenza patients and sepsis comparators grouped by testing rate.*

|  | N (%of population) | N with diabetes diagnosed in 1^st^ year | Unadjusted Hazard Ratio (95% CI) | Adjusted Hazard Ratio (95% CI) | P value |
| --- | --- | --- | --- | --- | --- |
| Low testers | | | | | |
| Influenza | 11,453 (83.5) | 73(0.6) | 0.91 (0.69-1.18) | 0.96 (0.73-1.26)^a^ | 0.774 |
| Sepsis Comparators | 33,495(86.9) | 210 (0.6) | 1.0 | 1.0 |  |
| High testers | | | | | |
| Influenza | 2,257(16.4) | 120 (5.3) | 1.17 (0.94-1.46) | 1.17 (0.94-1.47)^b^ | 0.165 |
| Sepsis Comparators | 5,066 (13.1) | 226 (4.5) | 1.0 | 1.0 |  |

*Low tester= 0-1 tests for diabetes/year, High tester= >1 test for diabetes per year*

*a-Adjusted for Age at index date, gender and preexisting hypertension.*

*b- Adjusted for Age at index date, gender*
