# Supplementary material for: Incidence of diabetes mellitus following hospitalisation with influenza: a population-based cohort study in England
Source: BMJ Open. 2026 May 4;16(5):e115391. doi: 10.1136/bmjopen-2025-115391 (PMC13141003; doi:10.1136/bmjopen-2025-115391)
Supplement: Supplementary file 2 [file bmjopen-2025-115391.reviewer_comments.pdf]

PEER REVIEW HISTORY

BMJ Open publishes all reviews undertaken for accepted manuscripts. Reviewers are asked to complete a checklist review form and are provided with free text boxes to elaborate on their assessment. These free text comments are reproduced below.

ARTICLE DETAILS

Title (Provisional)

Incidence of Diabetes Mellitus Following Hospitalisation with Influenza: A population-based cohort study in England

Authors

Middleton, Sophie; McKeever, Tricia M; Grudzinska, Frances; Huang, Yue; Bolton, Charlotte E

VERSION 1 - REVIEW

**Reviewer**	**1**
**Name**	**Dack, Kyle**
**Affiliation**	**University of Bristol, MRC Integrative Epidemiology Unit**
**Date**	**20-Jan-2026**
**COI**	**None**

Review summary:

The reporting of the methods and results adheres to best practices, and the interpretation is supported by the results. However, I have two concerns regarding the statistical analysis: (1) The covariate selection strategy has limitations and I am unsure of what benefits it adds, compared to full adjustment of potential covariates, and (2) there is an assumption that missing data is missing at random, and this was not really tested or justified. I have given suggestions on these in the more detailed comments below.

Abstract:

1. In the first result, it may aid readers to make the comparison more explicit, i.e. that the aHR is for those who were hospitalized with influenza.

Introduction:

2. The introduction is clear, giving a strong rationale for the study. However, the aim is quite brief, and may not fully prepare readers for the subsequent methods. I suggest possibly expanding it to clarify that it will be achieved by estimating incidence, risk compared to controls and sepsis patients, and by modelling risk factors.

Methods:

3. The three populations are described clearly and the criteria seem reasonable. However, what was the rationale for the second comparator group (sepsis patients)? Sepsis was not mentioned in the introduction, so I suggest clarifying the rationale. This would help readers to interpret the results.

4. The authors identified 13 potential confounders. 2 were “firm” confounders, adjusted in the model I assume based on prior evidence/theory. The other 11 were “uncertain” confounders, and were only included if they changed the HR by 5% or more. I am unsure regarding this, because (a) confounders could fail to change the HR, but if it is a true confounder it is still better to adjust for, to minimize bias (including in the 95% CI), and (b) this approach tests confounders individually, but there could be a cumulative effect from multiple confounders which fail to meet the 5% threshold.

-I am unclear of the benefit of this approach, rather than adjusting for all potential confounders. In general when evaluating “uncertain” confounders, assuming that a variable is not a confounder is a stronger assumption than assuming it is a confounder (https://pmc.ncbi.nlm.nih.gov/articles/PMC6447501/). I would suggest at least that the authors consider a fully adjusted model as a sensitivity analysis, to see whether this noticeably impacted the results.

5. Critical care admission is listed as a potential confounder. How is this defined, does it refer to admission during the influenza admission? Wouldn’t this make it a mediator, because it occurs after the exposure (influenza admission) - which would make it unsuitable for adjustment (due to splitting the causal path)?

6. Imputation: I recommend adding details of the package used (MICE?), which variables were used in the imputation (e.g. all variables, or excluding some), and iterations.

Results:

7. Missingness at random assumes that the probability of missing data is uncorrelated with the outcome. To test whether this holds, it would be valuable to compare the % missing data per variable stratified by outcome (DM vs no DM). If no differences are identified this would strengthen the justification for imputation – or if not, it would be an important limitation to acknowledge.

8. Table 3. Linked to the prior comment, the two models presented here have different adjustment sets. It does not seem plausible that something can be a confounder of influenza and DM, but not influence and prediabetes. It also hinders a comparison between these two sets of results, and if this covariate selection approach is retained I suggest a stronger justification for it.

9. Tables/figures/supplementary materials: All are clear, look to be consistent, and I have no other comments or suggestions. I also have no comments regarding the discussion - other than related to the two potential stats issues mentioned above.

**Reviewer**	**2**
**Name**	**Vyas , Arpita Kalla**
**Affiliation**	**Washington University in St Louis**
**Date**	**12-Feb-2026**
**COI**	**None**

This retrospective study examines the impact of an inpatient admission for influenza virus infection in the UK and the subsequent risk of developing diabetes mellitus. Although not novel, as there have been other studies addressing the association of influenza with the development of diabetes mellitus (PMID 36857969, PMID: 33660948, PMID: 29934759), the study being based in a different geographical location contributes to the developing literature in the field. The strengths are the number of subjects and the use of two different control populations. Overall, the manuscript is well written.

Minor comments:

Abstract: In the participant section for the matched group, for age, etc., please add that they were recruited from an outpatient GP practice.

Introduction:

• Line 32: "Previous studies have postulated a link between pandemic influenza or a history of a preceding influenza-like illness and new onset type 1 diabetes in children16 and adults.17 18". Please add the reference PMID: 36857969 on the incidence of type 2 DM risk post-influenza and on other influenza-type 2 DM studies, as mentioned in the discussion, to set the stage.

Methods:

Was ethical approval for this study obtained, or was it exempt from ethical review? Please clarify in your methods section.

Supplementary Figure 1 flowchart should be included in the main manuscript.

Were women with a previous history of gestational diabetes mellitus excluded from the total number?

Results:

Table 1: Seems like steroid use was higher in the influenza group compared to both sets of controls. Despite controlling for risk, DM was higher. It would be worth mentioning that steroid use was higher. It may be that the duration of steroid use in the influenza group differed as well, thereby increasing the risk of DM.

The unmatched control spent more time in the hospital, whereas Influenza patients were discharged earlier. Need to comment on this in your discussion.

Interestingly, chronic respiratory disease was higher in influenza compared to unmatched controls and could have played a role in increasing the incidence of DM.

Line 51: "After adjustment for confounders, the risk of developing any DM remained significantly higher in the influenza population (1.54 (95%CI 1.39-1.70, p<0.001))." Can you please clarify who the controls were here? Presuming NH control as listed in Table 2.

VERSION 1 - AUTHOR RESPONSE

Reviewer: 1
Dr. Kyle Dack, University of Bristol
Comments to the Author:
Review summary:
The reporting of the methods and results adheres to best practices, and the interpretation is supported by the results. However, I have two concerns regarding the statistical analysis: (1) The covariate selection strategy has limitations and I am unsure of what benefits it adds, compared to full adjustment of potential covariates, and (2) there is an assumption that missing data is missing at random, and this was not really tested or justified. I have given suggestions on these in the more detailed comments below.
Thank you for your detailed review and response. We hope we have answered your specific questions adequately below.

Abstract:
1. In the first result, it may aid readers to make the comparison more explicit, i.e. that the aHR is for those who were hospitalized with influenza.

We have amended the first line of the results in the abstract on page 2 to read *'Incidence of DM was 12.5 per 1000 person years. Adjusted hazard ratios (aHR) for developing DM after hospitalised influenza compared to matched controls was 1.54 (95% CI 1.39-1.70, p<0.001) and to hospitalised sepsis comparators 1.14 (95% CI 1.03-1.26, p=0.013).'* for clarity.

Introduction:
2. The introduction is clear, giving a strong rationale for the study. However, the aim is quite brief and may not fully prepare readers for the subsequent methods. I suggest possibly expanding it to clarify that it will be achieved by estimating incidence, risk compared to controls and sepsis patients, and by modelling risk factors.

We have amended the final sentence of the introduction on page 4 to expand on the aims of the study as suggested. It now reads: '*This study aimed to estimate the incidence and severity of diabetes mellitus following hospitalisation with influenza in England, to compare this risk with non-hospitalised controls and with patients hospitalised with sepsis and to identify associated risk factors.'*

Methods:
3. The three populations are described clearly and the criteria seem reasonable. However, what was the rationale for the second comparator group (sepsis patients)? Sepsis was not mentioned in the introduction, so I suggest clarifying the rationale. This would help readers to interpret the results.

Thank you for this observation. We wanted to choose a hospitalised patient cohort to eliminate the effect that hospitalisation could have on a patient developing DM. We also wanted to choose an inflammatory condition due to the proposed mechanisms of development of diabetes mellitus that would be a similar demographic to influenza patients, thus choosing hospitalised sepsis patients. We have clarified this on page 6 in the 'Control and comparator groups' in the methodology section. We spent a lot of time discussing the optimal comparator group and acknowledge that there is not a single ideal.

4. The authors identified 13 potential confounders. 2 were "firm" confounders, adjusted in the model I assume based on prior evidence/theory. The other 11 were "uncertain" confounders and were only included if they changed the HR by 5% or more. I am unsure regarding this, because (a) confounders could fail to change the HR, but if it is a true confounder it is still better to adjust for, to minimize bias (including in the 95% CI), and (b) this approach tests confounders individually, but there could be a cumulative effect from multiple confounders which fail to meet the 5% threshold.
-I am unclear of the benefit of this approach, rather than adjusting for all potential confounders. In general when evaluating "uncertain" confounders, assuming that a variable is not a confounder is a stronger assumption than assuming it is a confounder (https://pmc.ncbi.nlm.nih.gov/articles/PMC6447501/). I would suggest at least that the authors consider a fully adjusted model as a sensitivity analysis, to see whether this noticeably impacted the results.

Co-variates were chosen as variables that could potentially influence whether someone developed diabetes based on prior knowledge. The reason that the method of only including the covariates that had an effect on the model (changed HR by at least 5%) was used was to create the simplest model possible that adjusted for confounders. Adding too many variables to a model can cause issues such as overfitting the model, reducing power and precision, reducing the degrees of freedom and increasing the size of standard errors.

To answer your query, the adjusted hazard ratios for development of diabetes and prediabetes in influenza patients compared to non-hospitalised controls and hospitalised sepsis patients (Table 2&3), were fully adjusted with all co-variates. The co-variates included were age at admission, gender, BMI, IMD level, ethnicity, smoking status at admission, pre-existing hypertension, hyperlipidaemia, chronic lung disease and high risk of diabetes, influenza vaccination status in year prior to admission and oral corticosteroid prescription in the year prior to admission and during follow up. In hospitalised patients we also adjusted for critical care admission. A similar pattern was seen in these fully adjusted models and so we have chosen to keep the most simplified adjusted model for the reasons stated above in the paper. We have not put the fully adjusted models in the paper.

5. Critical care admission is listed as a potential confounder. How is this defined, does it refer to admission during the influenza admission? Wouldn't this make it a mediator, because it occurs after the exposure (influenza admission) - which would make it unsuitable for adjustment (due to splitting the causal path)?

Critical care admission data is provided by HES and they define it as an admission to either an Intensive Care Unit (ICU) or High Dependency Unit (HDU) during the inpatient stay. We have used it in our study as a marker for severity of the influenza infection and so a potential confounder.

6. Imputation: I recommend adding details of the package used (MICE?), which variables were used in the imputation (e.g. all variables, or excluding some), and iterations.

Missing data was only present in patient smoking status, BMI, ethnicity and IMD level therefore these were the only variables used in the imputation. I have included this on page 8 in the statistical analysis section and added details of the MICE package.

Results:

7. Missingness at random assumes that the probability of missing data is uncorrelated with the outcome. To test whether this holds, it would be valuable to compare the % missing data per variable stratified by outcome (DM vs no DM). If no differences are identified this would strengthen the justification for imputation – or if not, it would be an important limitation to acknowledge.

Thank you for this query. To assess whether data was missing at random we first analysed the relationship between missing variables in each patient. As shown below (Figure 1) most people only had one missing variable supporting missingness at random rather other issues related to their GP record.

Figure 1: The relationship between missing variables in the hospitalised influenza cohort.

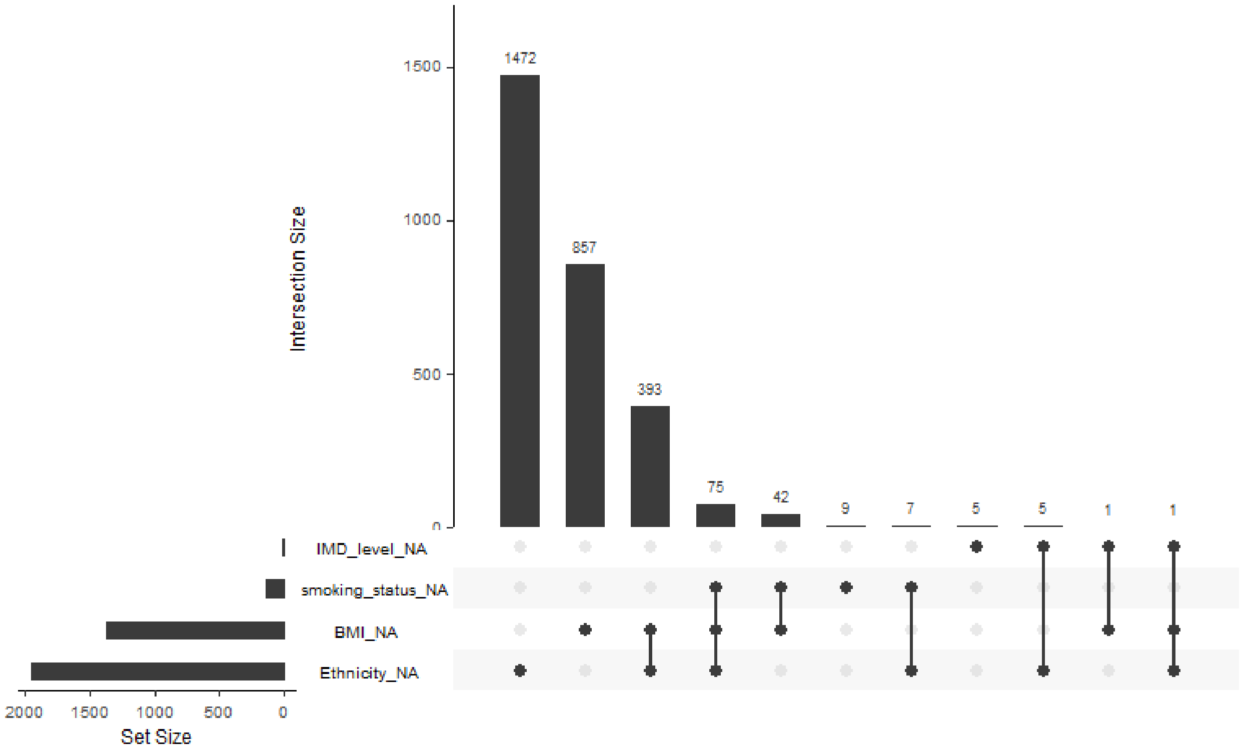

We then compared the percentage of missing data in the influenza cohort by whether they were diagnosed with diabetes or not and used chi squared testing to check significance (Table 1). There were more missing data present in influenza patients who were not diagnosed with DM, especially among BMI and ethnicity. This may reflect healthcare utilisation or GP record quality of the individual. However, in previous sensitivity analysis into primary care diabetes testing rate and diabetes diagnosis, Influenza patients were more likely to be diagnosed than NH controls even among low testers, i.e. lower healthcare users (Supplementary Table 4). We have acknowledged this as a limitation in our study in the discussion on page 16.

Table 1: Percentage of missing data in each variable stratified by outcome

	**Diagnosed with Diabetes (n=610)**	**No Diabetes (N=13,100)**	**P Value from Chi Squared test**
	Number with missing data (%)	Number with missing data (%)	
**Any missing data**	79 (13.0)	2,788 (21.3)	<0.001
**Ethnicity**	57 (9.3)	1,890 (14.5)	<0.001
**IMD level**	0	12 (0.1)	0.96*
**BMI**	34 (5.6)	1,335 (10.2)	<0.001
**Smoking status**	0	133 (1.0)	0.02*

** Yates continuity correction used*

8. Table 3. Linked to the prior comment, the two models presented here have different adjustment sets. It does not seem plausible that something can be a confounder of influenza and DM, but not influence and prediabetes. It also hinders a comparison between these two sets of results, and if this covariate selection approach is retained I suggest a stronger justification for it.

We have adjusted the hazard ratio models as described above in question 4. In each model only those covariates that notably changed the model were included. The prediabetes population was slightly different compared to the diabetes population analysed because we excluded those that had a pre-existing diagnoses of prediabetes as well as diabetes. There may also be a difference in the profile of a person who just develops prediabetes instead of diabetes mellitus, but we have not analysed this further.

9. Tables/figures/supplementary materials: All are clear, look to be consistent, and I have no other comments or suggestions. I also have no comments regarding the discussion - other than related to the two potential stats issues mentioned above.

Reviewer: 2
Dr. Arpita Kalla Vyas , Washington University in St Louis
Comments to the Author:
This retrospective study examines the impact of an inpatient admission for influenza virus infection in the UK and the subsequent risk of developing diabetes mellitus. Although not novel, as there have been other studies addressing the association of influenza with the development of diabetes mellitus (PMID 36857969, PMID: 33660948, PMID: 29934759), the study being based in a different geographical location contributes to the developing literature in the field. The strengths are the number of subjects and the use of two different control populations. Overall, the manuscript is well written.

Thank you for your review, we hope we have addressed your comments adequately below.

Minor comments:
10. Abstract: In the participant section for the matched group, for age, etc., please add that they were recruited from an outpatient GP practice.

We have amended the abstract for clarity as per your suggestion. It now reads *"A randomly selected group a) from CPRD records matched for age, sex and GP practice and b) an un-matched group of hospitalised sepsis patients were used as comparator groups."*

Introduction:
11. Line 32: “Previous studies have postulated a link between pandemic influenza or a history of a preceding influenza-like illness and new onset type 1 diabetes in children16 and adults.17 18”. Please add the reference PMID: 36857969 on the incidence of type 2 DM risk post-influenza and on other influenza-type 2 DM studies, as mentioned in the discussion, to set the stage.

Introduction on page 4 has been amended as per your suggestions to include studies mentioned in the discussion.

Methods:
12. Was ethical approval for this study obtained, or was it exempt from ethical review? Please clarify in your methods section.

We have included an Ethics statement at the end of the paper on page 17. It reads:

‘***Ethics approval: CPRD has ethics approval from the UK’s Health Research Authority (HRA) Research Ethics Committee (REC) (East Midlands – Derby, 05/MRE04/87). No additional ethics approval is required for observational studies using CPRD Aurum data for public health research, subject to individual research protocols meeting CPRD data governance requirements. Approved study protocol # 22_002065 available on request.’***

13. Supplementary Figure 1 flowchart should be included in the main manuscript.

We have included this in the manuscript as per your recommendation on page 9.

14. Were women with a previous history of gestational diabetes mellitus excluded from the total number?

Previous gestational diabetes was not a reason to be excluded. It was however used as a risk factor for developing DM in the ‘at risk of diabetes’ covariate. See the covariate definition section of the methods on page 6-7 for full details.

Results:
15. Table 1: Seems like steroid use was higher in the influenza group compared to both sets of controls. Despite controlling for risk, DM was higher. It would be worth

mentioning that steroid use was higher. It may be that the duration of steroid use in the influenza group differed as well, thereby increasing the risk of DM.

Steroid use was calculated from prescriptions of oral corticosteroids in general practice the year prior to hospitalisation and during follow up. We do not have access to information on steroid use during hospitalisation. For the outcome measure of diabetes mellitus, our adjusted hazard ratios have also been adjusted for steroid prescription (Table 2 and 3).

16. The unmatched control spent more time in the hospital, whereas Influenza patients were discharged earlier. Need to comment on this in your discussion.

We have added a section in the baseline characteristics on page 8 noting the important differences between our cases and comparator groups. We have also added a sentence in the limitations section of the discussion as suggested regarding the differences between cohorts.

17. Interestingly, chronic respiratory disease was higher in influenza compared to unmatched controls and could have played a role in increasing the incidence of DM.

We agree that this is an interesting point, perhaps linked to historical steroid usage, we have also adjusted for chronic lung disease in our adjusted hazard ratios.

18. Line 51: "After adjustment for confounders, the risk of developing any DM remained significantly higher in the influenza population (1.54 (95%CI 1.39-1.70, p<0.001))." Can you please clarify who the controls were here? Presuming NH control as listed in Table 2.

We have clarified this now. Sentence now reads: *"After adjustment for confounders the risk of developing any DM compared to NH controls remained significantly higher in the influenza population (1.54 (95%CI 1.39-1.70, p<0.001))."*

Reviewer: 1
Competing interests.: NA

Reviewer: 2
Competing interests.: Not applicable

VERSION 2 - REVIEW

**Reviewer**	**1**
**Name**	**Dack, Kyle**
**Affiliation**	**University of Bristol, MRC Integrative Epidemiology Unit**
**Date**	**25-Mar-2026**
**COI**	

Thank you to the authors for their responses to my queries, and the clarifications/additions to the manuscript.
